# Altered network organization with shift to local processing during adaptive behaviour in multiple sclerosis

**DOI:** 10.1093/braincomms/fcag397

**Published:** 2026-10-19

**Authors:** Julia Elmers, Moritz Mückschel, Tjalf Ziemssen, Christian Beste

**Affiliations:** Cognitive Neurophysiology, Department of Child and Adolescent Psychiatry, Faculty of Medicine, TU Dresden, 01309 Dresden, Germany; Center of Clinical Neuroscience, Department of Neurology, University Hospital Carl Gustav Carus, TU Dresden, 01309 Dresden, Germany; Cognitive Neurophysiology, Department of Child and Adolescent Psychiatry, Faculty of Medicine, TU Dresden, 01309 Dresden, Germany; Center of Clinical Neuroscience, Department of Neurology, University Hospital Carl Gustav Carus, TU Dresden, 01309 Dresden, Germany; Cognitive Neurophysiology, Department of Child and Adolescent Psychiatry, Faculty of Medicine, TU Dresden, 01309 Dresden, Germany; German Center for Child and Adolescent Health (DZKJ), TU Dresden, 01309 Dresden, Germany

**Keywords:** directed connectivity, neural oscillations, theta band, task switching, multiple sclerosis

## Abstract

To adjust to changing environmental demands, the brain needs to organize and (re)configure mental resources in so-called task sets. Following a network perspective of cognitive functions, these processes are considered to rely on dynamic exchange of information between distant brain regions, possibly synchronized by slow-wave theta oscillations. However, few attempts have been made to describe the directional transfer of information in theta networks during behavioural adaptation. In the current study, we examined directed neural information transfer in 57 healthy individuals and 57 patients with multiple sclerosis with a focus on brain–behaviour associations during task switching. For this, we calculated behavioural switch costs and further applied an artificial neural network-based approach on neurophysiological EEG data to estimate linear and non-linear effective connectivity measures within cortical networks during task switching. Source reconstruction and connectivity analyses revealed distinct neural network architectures and communication strategies during task switching in healthy controls compared to patients with multiple sclerosis. In healthy controls, enhanced modulations of long-range fronto-parietal connectivities were associated with higher reaction time switch costs, possibly reflecting increased cognitive demands rather than adaptive processes. In contrast, patients with multiple sclerosis relied more strongly on local intra-regional modulations, which is consistent with the idea of a dysconnectivity syndrome in multiple sclerosis. Strikingly, stronger modulations in self-connectivities were associated with better accuracy measures in patients with multiple sclerosis, suggesting a compensatory mechanism to support performance. Notably, only non-linear directed connectivity measures were related to behavioural switch costs, emphasizing the importance of non-linear models for adaptive cognitive processes.

## Teaser

Elmers *et al.* report that healthy controls and people with multiple sclerosis (pwMS) show distinct neural network architectures and communication strategies during task switching. In particular, pwMS relied more strongly on local intra-regional modulations, which likely mirror a compensatory mechanism to support performance.

## Graphical Abstract

**Graphical Abstract:**
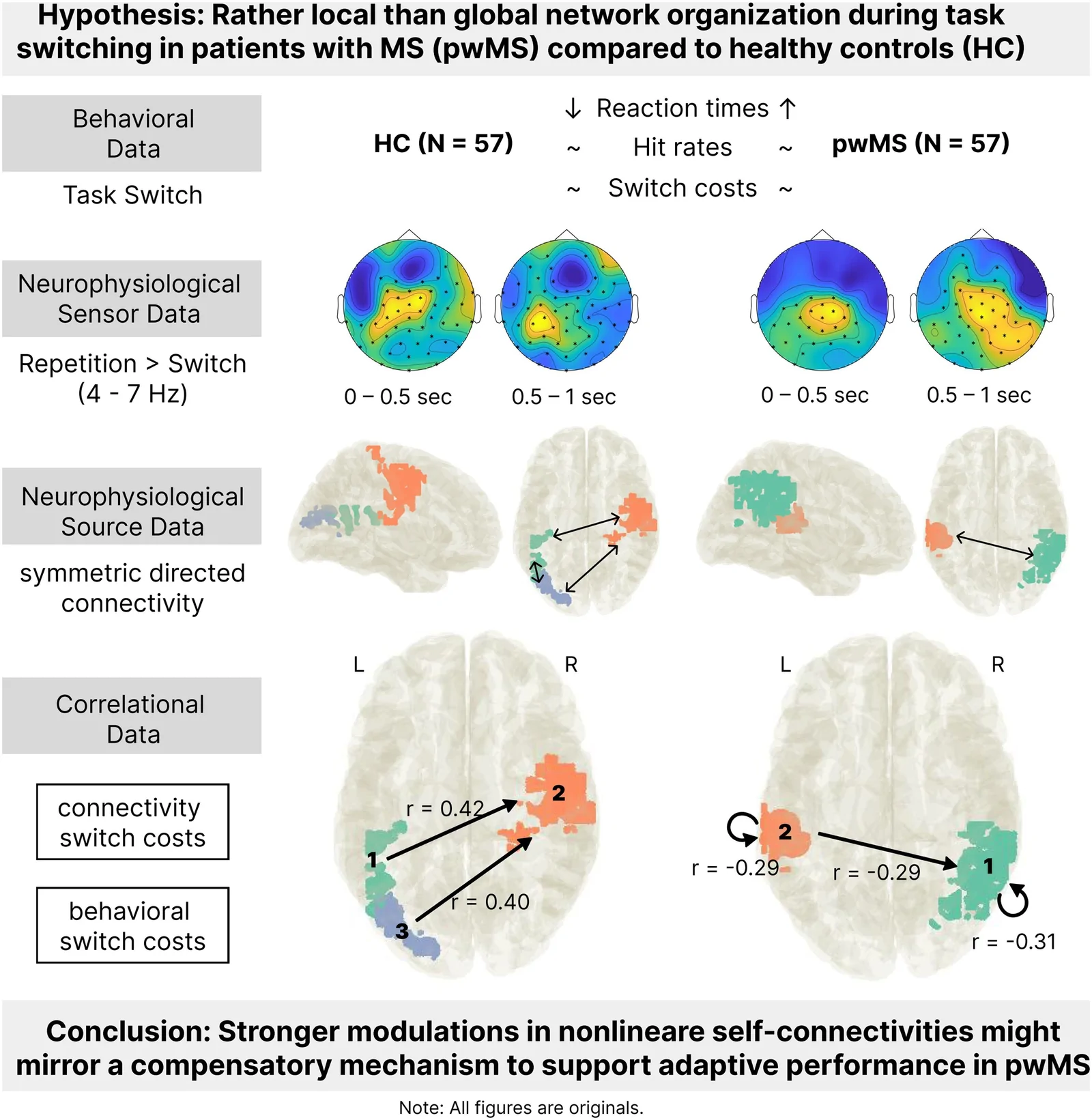

## Introduction

Adaptive behaviour relies on the brain’s ability to adjust to changing environmental demands. To enable such flexibility, the brain organizes and configures mental resources in so-called (procedural) task sets, where information regarding stimulus features and responses to these stimuli is combined (task set configuration) and stored in memory.^1,2^ In a changing environment, non-appropriate task sets need to be rapidly reconfigured to allow goal-directed behaviour.^1^ The processes of binding and retrieving of stimulus-response features are likely supported by slow-wave theta band (4–7 Hz) oscillations in the brain,^3,4^ reflecting their importance in cognitive control processes, particularly in conditions of increased mental effort or conflict.^5^ Importantly, theta is not only involved in stimulus-response binding and cognitive flexibility^6-9^ but also in the synchronization of brain-wide regional activity facilitating efficient network organization and cognitive control.^5,10,11^

Modern cognitive neuroscience increasingly recognizes that such control functions arise not from isolated regions, but from interactions within and between large-scale brain networks^12^ like the fronto-parietal or the salience network.^13^ This ‘network perspective’ is supported by findings indicating that long-range rather than short-range structural connections are strongly related to decreases in network efficiency and global cognitive impairment,^14^ and that increased functional connectivity between fronto-parietal regions (i.e. within the cognitive control network) is causally associated with better cognitive performance.^15,16^ Especially fronto-parietal regions together with cingulo-opercular regions and the insular cortex are consistently reported to be of importance for adaptive behaviour.^17-20^ Importantly, these areas are also associated with cognitive control processes supported by theta band synchronization.^5,21,22^

In the clinical field, the network perspective is especially relevant for diseases of the central nervous system such as multiple sclerosis, a chronic autoimmune disorder characterized by demyelination of cortical axons.^23^ In this disease, long-range white matter tracts in particular are disrupted, leading to reduced structural and functional connectivity between distant regions and a compensatory shift towards local processing.^24-26^ On a behavioural level, people with multiple sclerosis (pwMS) often show worse performance in a wide range of cognitive functions^27,28^ due to progressive neurodegeneration.^29^ This degenerative progress inevitably leads to a network disruption, which is why multiple sclerosis is often described as a ‘dysconnectivity syndrome’,^30,31^ where reduced functional and effective connectivity correlate with poorer cognitive performance.^32-35^ In the same vein, recent findings on effective connectivity during complex walking tasks, another domain often impaired in multiple sclerosis, showed clear connectivity differences between healthy controls (HCs) and pwMS.^36,37^ While HC showed bilateral activation in balanced widespread networks, pwMS showed left-localized, more regional activation and an overall reduced network efficiency. Importantly, altered connectivity in multiple sclerosis may not exclusively reflect pathological breakdown. In some cases, changes in network architecture may represent compensatory mechanisms, which initially support adaptive functioning but may deteriorate with disease progression.^24-26^

To explore underlying network dynamics of cognitive flexibility more precisely, the present study investigates directed information transfer during adaptive behaviour in pwMS and HC, focusing on theta-modulated network organization given its importance for network synchronization. Traditionally, research has focused primarily on linear network interactions, but recent findings show the importance of non-linear models,^38-40^ which seem to have a greater sensitivity to biologically plausible mechanisms (e.g. dynamic adjustments, non-additive influences, or feedback loops) underlying flexible cognition, as shown previously.^41^ Prior functional MRI research has shown that directed network interactions play a key role when individuals need to switch between tasks.^42-44^ However, it remains unclear how theta-modulated directional communication contributes to task-switching performance, especially in clinical populations. To investigate directed communication during task switching in pwMS and HCs, we applied the non-linear Causal Relationship Estimation by Artificial Neural Network (nCREANN) approach^45^ to examine specific profiles of directed linear and non-linear connectivities in theta band activity during task switching. This approach has shown before that directed connectivity during several perception-action integration processes is relevant to facilitate goal-directed behaviour.^46-48^

The current study uses a modified task-switching paradigm^49^ to vary the cognitive load of task set retrieval and reconfiguration during adaptive behaviour.^50^ This so-called memory-based task switching relies to a greater extent on working memory processes,^51-53^ which are also strongly influenced by theta modulations.^54,55^ We hypothesize that groups will differ in network organization and efficiency, whereby pwMS will likely rely on a more local than global network organization due to the nature of the disease. Following from this, we would also expect pwMS to show poorer behavioural performance [i.e. like longer reaction times (RTs) and worse accuracy measures] due to network inefficiency. Further, by comparing behavioural switch costs (i.e. performance differences between switch and repetition trials), which are considered to mirror time-consuming task set reconfiguration processes,^1,56,57^ we aim to determine how differences in network efficiency and organization underlie adaptive behaviour between pwMS and HC.

## Materials and methods

### Participants

A total of 125 participants were recruited for the current study, including 57 pwMS (37 female; mean age = 36.4 ± 7.8 years) and 68 HCs (47 female; mean age = 34.6 ± 9.9 years). Six healthy participants had to be excluded due to missing or bad data sets. To enhance comparability between groups (as pwMS were significantly older), we excluded five healthy individuals blind to results based on their age and sex, resulting in a final sub-sample size of 57 HCs (37 female; mean age 35.2 ± 9.2 years). Further, due to a missing logfile of one healthy individual, the behavioural data analysis was conducted with a sample of *N* = 113 (HC = 56; pwMS = 57) participants. Although data from the HC group partially overlaps with a previous study (not published yet), the current sample differs substantially due to the matching procedure.

All participants were right-handed, had normal or corrected-to-normal vision and hearing and reported no history of psychiatric or (other) neurological conditions. Informed written consent was obtained from all individuals prior to participation. The study was approved by the Ethics Committee of TU Dresden (EK 219062018) and conducted in accordance with the Declaration of Helsinki.

Disease duration was calculated for pwMS from the date of multiple sclerosis diagnosis to the date of study participation (month and year). The average duration of multiple sclerosis was 30.4 ± 41.7 months (2.5 ± 3.5 years). In the current pwMS sample, the disease duration varied greatly (0–192 months; 0–16 years) with a median duration of 11 months.

Regarding clinical measures of disability, the Expanded Disability Status Scale (EDSS) was used to examine eight functional systems: pyramidal (e.g. paralysis), cerebellar (e.g. ataxia or tremor), brainstem (speech or swallowing disorders), sensory, bowel and bladder, visual, cerebral (e.g. cognition) and other.^58^ Thereby, an EDSS score of 0 indicates a normal neurological exam, whereby EDSS scores from 1.0 to 4.5 indicate (minimal) signs of disability in one or more functional system(s) with no impairment regarding ambulation. In the current sample, pwMS had a median EDSS of 2 (interquartile range = 1.5–3.0) with EDSS values ranging from 0 to 4.5.

### Task and procedure

A task-switching paradigm was employed to investigate memory-based task switching (Fig. 1). The paradigm can also be used to evaluate cue-based task switching.^52^ However, in the current study, we focused on the more demanding version only considering memory-based trials. We used data of six memory-based blocks with a total of 198 trials, consisting of 66 switch trials and 132 repetition trials due to a fixed stimulus order and switching frequency of 33.3%, respectively. A (dummy) cue was shown at the start of each trial 1300 ms prior to the target’s appearance, and it stayed visible until the target stimulus was shown. Trials were considered missed if there was no reaction within 2500 ms after target presentation. Participants saw a dark screen 500 ms after each response. After that, they received feedback for 500 ms in the form of a ‘+’ or ‘−’ sign. This was followed by a jittered feedback-cue interval, which resulted in a total response-cue interval of about 2000 ms (see Fig. 1).

**Figure 1:**
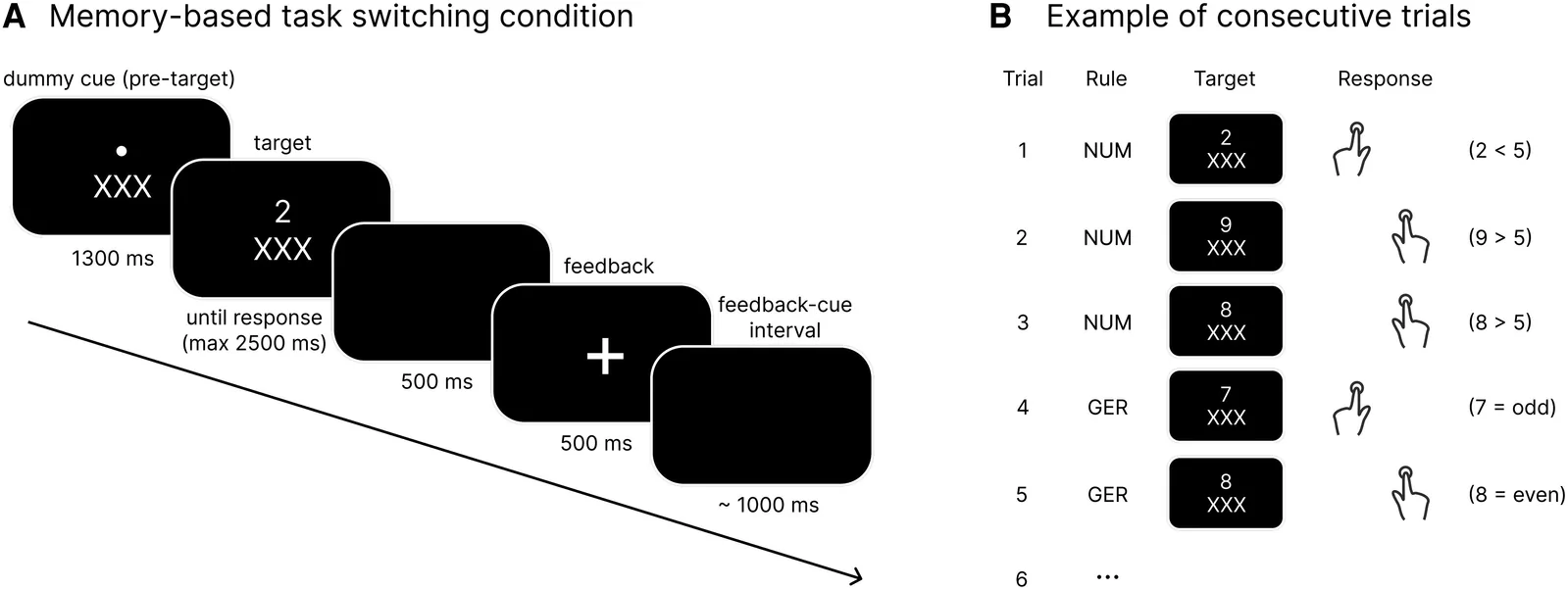
**The task switch paradigm**. (**A**) An illustration of one possible trial in the memory-based condition of the task switch paradigm. (**B**) A schematic representation of five consecutive trials with a fixed order of response rules.

The target stimuli were digits ranging from 1 to 9 (excluding 5), presented in white font against a black background on a computer screen. The paradigm involved three task rules. Depending on the rule, participants had to decide whether the digit was smaller or greater than 5 (‘NUM’ for numeric), even or odd (‘GER’ for even) or whether it was shown in large or small font size (‘SG’ for font size). In the memory-based block, participants had to mentally track a fixed sequence of task rules. The order of rules was always the same (three trials NUM, three trials GER and three trials SG rule (NUM, NUM, NUM, GER, GER, GER, SG, SG, SG, NUM, NUM, NUM, …). A detailed description of the task design and procedure can be found in the Supplementary material.

### EEG recording and preprocessing

EEG data were collected using a 60-channel Ag/AgCl electrode cap, with electrodes evenly distributed across the scalp. The data were sampled at 500 Hz using a QuickAmp or BrainAmp DC/ExG amplifier (Brain Products GmbH, Gilching, Germany) and downsampled to 256 Hz and bandpass filtered between 0.5 and 40 Hz. To minimize noise and artefacts, a multi-step cleaning procedure was applied in a preprocessing pipeline with Brain Vision Analyzer (Brain Products GmbH). First, a manual artefact rejection was conducted to remove technical or random artefacts, such as flat or noisy channels, power spikes and muscle tension. Next, an independent component analysis was used to identify and eliminate recurring artefacts, including eye blinks, horizontal eye movements and pulse artefacts. A second round of manual artefact rejection was performed if necessary. Channels that had been removed during visual inspection were subsequently back-interpolated. Following preprocessing, the EEG data were segmented into epochs time-locked to the target onset. Each epoch encompassed a 4-s window, covering 2000 ms before and 2000 ms after target onset.

### EEG time-frequency analysis and source localization pipeline

Time-frequency analysis and source localization were conducted using a pipeline previously applied in cognitive control tasks.^41,46^ All analyses were performed in MATLAB using the FieldTrip toolbox.^59^

Time-frequency representations were computed for EEG data across a time window around one before and after stimulus onset to assess spectral power differences in the theta frequency band for both conditions. To localize the underlying sources of theta oscillations, the dynamic imaging of coherent sources (DICS) beamformer^60^ was applied using a forward model based on the Montreal Neurological Institute brain template. DICS estimates frequency-specific source activity based on a sensor-level cross-spectral density (CSD) matrix and is known for its high localization accuracy in oscillatory data.^61,62^ Theta band activity was analysed in a post-stimulus time window of 0–750 ms, defined based on prior time-frequency results. A spectral analysis to compute the power values and the CSD matrix was conducted using a Hanning taper. EEG electrodes were aligned to the forward model, and the brain volume was segmented into a 5 mm grid. Leadfield matrices were computed for each voxel, and a common spatial filter (5% regularization) was applied across conditions. To correct for central-head noise bias, source estimates were normalized using the smallest eigenvalue of the CSD matrix.^63^

In the next step, the top 3% of active voxels within pre-defined anatomical regions were identified and grouped using the Density-Based Spatial Clustering of Applications with Noise (DBSCAN) algorithm,^64,65^ yielding spatially defined activity clusters (ACs). Cluster sizes in HCs were similar in AC1 and AC3 in both conditions (115/116 voxels for AC1 and 45/48 voxels for AC3), while the number of voxels differed more pronounced in AC2 (93 in repetition, 186 in switch trials). In pwMS, cluster sizes also differed slightly in both ACs (255/230 voxels for AC1 and 49/74 voxels for AC3). Thereby, an epsilon value of 1.5 times the grid spacing was used to allow grouping of edge-adjacent voxels. Voxels were labelled according to the automated anatomical labelling atlas.^66^

Finally, virtual neural time series were reconstructed for each voxel in each AC using the linearly constrained minimum variance (LCMV) beamformer.^63^ Time series were bandpass filtered in the theta frequency band, and voltage values were averaged across voxels for each AC and condition.

### Directed connectivity analysis

Directed connectivity analyses were performed on post-target EEG data filtered in the theta frequency band. Preprocessed EEG signals were bandpass filtered using a Hamming windowed sinc finite impulse response (FIR) filter to isolate theta band activity while preserving the temporal characteristics of the original signal. Filtered time series were then segmented and sorted according to experimental condition (repetition versus switch).

Directed interactions among ACs (which have been identified by the DICS/DBSCAN pipeline) were assessed using the nCREANN algorithm.^45^ This machine learning-based method estimates directed functional connectivity by combining a non-linear multivariate autoregressive model with an artificial neural network, allowing it to capture both linear and non-linear signal dynamics. This approach enables the evaluation of directed information flow across distributed brain regions, surpassing the limitations of traditional linear multivariate autoregressive methods.^41,46,48,67,68^

For each subject, single-trial reconstructed source time series (i.e. previously derived with the LCMV beamformer) were concatenated to ensure sufficient data length for model estimation. The optimal model order *P* was determined individually using the Akaike and Schwarz information criteria^69^ and applied separately for each condition and subject. The curves were averaged across all subjects within one group, and the mean ± standard error was plotted for each condition. Model order selection was then made through visual inspection of these curves and did not differ between groups (*P* = 7 for HC; *P* = 7 for pwMS).

To facilitate inter-subject comparisons, resulting connectivity values were normalized within a [0,1] range by dividing each subject’s connectivity values by their respective maximum value across conditions, computed separately for linear and non-linear estimates. All network connectivity patterns represent meaningful findings as nCREANN uses surrogate data with 100 permutations to correct for multiple comparisons.

### Statistical analyses

We used IBM SPSS Statistics version 29.0.0.0 for behavioural data analyses. Averaged RTs and hit rates for repetition and switch trials were calculated for each participant, yielding the factor combinations of memory repetition and memory switch. We used 2 × 2 mixed ANOVAs with the within-factor ‘switching’ and between-factor ‘group’ to investigate differences in repetition and switch trials between groups (HC versus multiple sclerosis) for RTs and hit rates. Furthermore, to evaluate differences within groups, we used *post hoc* paired-sample *t*-tests with Bonferroni correction.

Moreover, we computed ‘switch costs’ by subtracting performance measures of repetition trials from switch trials (for RTs and hit rates, as well as for connectivity measures) and used Welch’s one-way ANOVAs to check for significant differences between groups. Finally, Pearson’s linear correlation analyses were performed between behavioural outcomes and connectivity measures (i.e. nCREANN results). To correct for multiple comparisons, we used the false discovery rate (FDR) correction. For the evaluation of EEG sensor data of EEG, we used cluster-based permutation tests to account for Type I errors (i.e. to correct for multiple comparisons).

## Results

### Behavioural results

Results of behavioural analyses are shown in Fig. 2.

**Figure 2:**
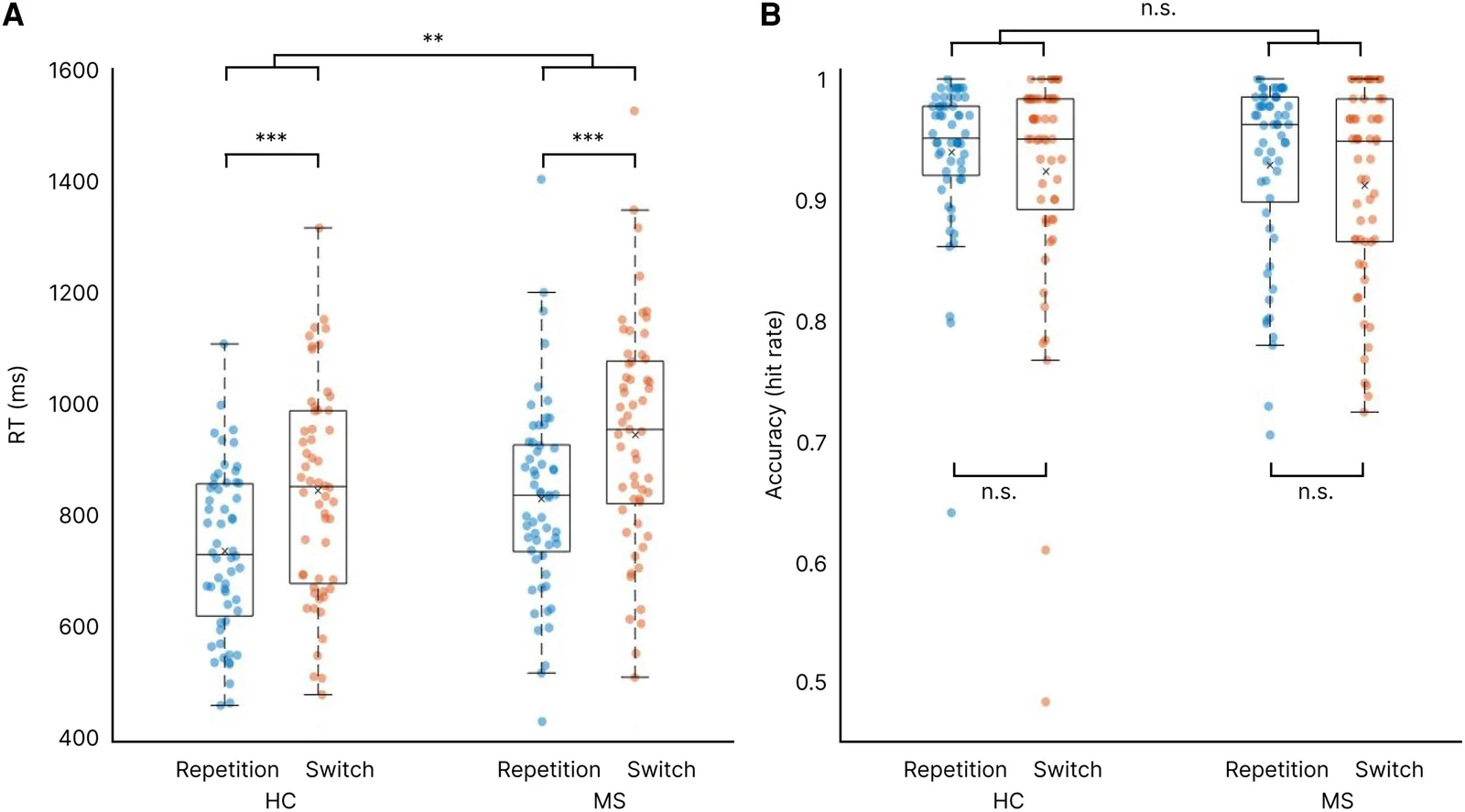
**Boxplots of behavioural results from the task switch paradigm for both groups**. Shown are (**A**) RTs and (**B**) hit rates for the memory-based trials. For RTs, we found a main effect of ‘group’ (higher RTs in pwMS versus HC) and a main effect for ‘switching’ (higher RTs in switching versus repetition). For hit rates, a main effect for ‘switching’ (lower hit rates in switching versus repetition). We used 2 × 2 mixed ANOVAs to test differences between groups and *post hoc* paired-sample *t*-tests (Bonferroni corrected) to test differences within groups (*N* = 113; HC = 56, pwMS = 57). *Post hoc* paired-sample *t*-tests revealed significant higher RTs in switch compared to repetition trials in both groups (HC: *P* = 6.36 × 10^−13^, *P*_corr_ < 0.001; pwMS: *P* = 2.73 × 10^−14^, *P*_corr_ < 0.001). Paired-sample *t*-tests of hit rates within groups revealed no differences within groups between switch and repetition trials (HC: *P* = 0.072, *P*_corr_ = 0.072; pwMS: *P* = 0.056, *P*_corr_ = 0.056). The main effects of switching are not shown due to difficulties in graphical representation. ***P < 0.001, **P < 0.05, n.s. = non-significant. Centreline = median; × = mean; box limits = upper and lower quartiles, whiskers = minimum and maximum within 1.5× interquartile range, dots = individual data points.

Regarding RTs, the mixed ANOVA showed a main effect of ‘group’ (HC versus multiple sclerosis) for repetition [*F*(1,111) = 9.021; *P* = 0.003; *η*^2^ = 0.0 75] and ‘switching’ (repetition versus switch) [*F*(1,111) = 142.481; *P* < 0.001; *η*^2^ = 0.562]. RTs of pwMS were significantly slower in both conditions (repetition, 831.51 ± 171.03 ms; switch, 945.18 ± 201.18 ms) compared to HCs (repetition, 738.33 ± 146.62 ms; switch, 845.10 ± 187.73 ms). We did not find an interaction effect of ‘group’ *×* ‘switching’ [*F*(1,111) = 0.140; *P* = 0.709]. Within the HC group, paired-sample *t*-tests with Bonferroni correction revealed significant higher RTs switch compared to repetition trials [*M*_Diff_ = 106.77; *P* < 0.001; 95% CI (80.78, 132.76)]. The same pattern was found in the group of pwMS [*M*_Diff_ = 113.68; *P* < 0.001; 95% CI (87.92, 139.44)].

For hit rates, there was a main effect of ‘switching’ [*F*(1,111) = 7.028; *P* = 0.009; *η*^2^ = 0.060], with lower hit rates in switching (91.7 ± 8.9%) compared to repetition (93.4 ± 6.9%). There was no significant main effect of ‘group’ [*F*(1,111) = 0.669; *P* = 0.415] nor an interaction effect [*F*(1,111) = 0.005; *P* = 0.946]. *Post hoc* paired-sample *t*-tests with Bonferroni correction revealed no significant differences of hit rates within groups (all *P* > 0.05; HC repetition: 93.9 ± 6.1% versus switch: 62.3 ± 9.6%; multiple sclerosis repetition: multiple sclerosis: 92.9 ± 7.6% versus switch: multiple sclerosis: 91.2 ± 8.1%).

Switch costs were calculated by subtracting performance measures in the repetition trials from switch trials. Switch costs of RTs did not differ between groups [HC, 106.77 ± 81.02 ms; multiple sclerosis, 113.68 ± 112.47 ms; *F*(1,112) = 0.140; *P* = 0.709], nor did accuracy switch costs [HC, −1.6 ± 7.8%; multiple sclerosis, −1.7 ± 5.5%; *F*(1,112) = 0.005; *P* = 0.746].

### Association of clinical parameters and behavioural results in people with multiple sclerosis

Correlational analysis between clinical parameters was applied for pwMS. Disease duration significantly correlated with RTs in both switch (*r* = 0.286; *P* = 0.031) and repetition (*r* = 0.372; *P* = 0.004) trials, and with hit rates in repetition trials (*r* = −0.386; *P* = 0.003), indicating slower RTs and worse accuracy with prolonged disease duration. However, we did not find a correlation between switch costs and disease duration (all *P* > 0.103).

For measures of neurological disability, higher EDSS scores were associated with lower accuracy measures in both experimental conditions (repetition: *r* = −0.586; *P* < 0.001; switch: *r* = −0.443; *P* < 0.001), but showed no significant correlation with RTs (all *P* > 0.053). Moreover, elevated EDSS scores were associated with higher RT switch costs (*r* = 0.341; *r* = 0.009), but not with accuracy switch costs (*P* = 0.264).

### Neurophysiological results of memory-based task switching

#### Sensor data

The results of the time-frequency analysis showed similar results for the HC and pwMS (Fig. 3) with cluster-based permutation tests revealing significant power effects between repetition and switch trials in the theta frequency band. Thereby, greater theta power was found on the sensor level in memory-repetition trials (i.e. positive clusters). A comparison between groups regarding task conditions (repetition and switch, respectively) did not show significant power differences for the theta frequency band (Fig. 3C).

**Figure 3:**
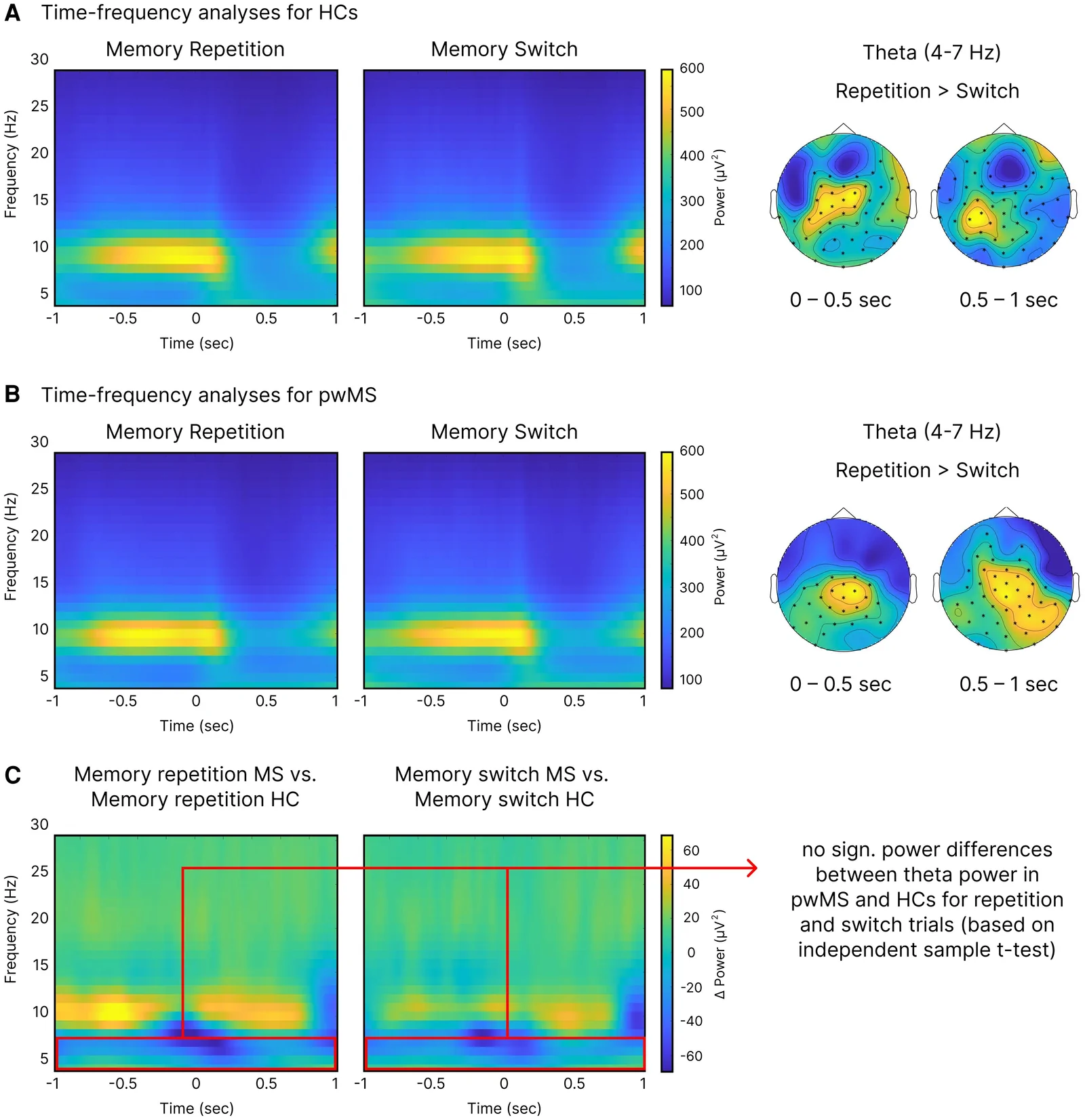
**Time-frequency results for HCs and pwMS**. (**A**) Results for HC in both conditions separately and the results from the cluster-based permutation test. (**B**) The same results for pwMS. All significant cluster-based permutation tests (marked with ‘*’) revealed positive clusters, indicating higher power values in repetition than switch trials. (**C**) Differences in power for repetition (left) and switch trials (right) between groups for all frequencies. There were no significant differences between theta power of the two groups during the time interval of interest [−1 1] based on results of independent sample *t*-test between HCs (*n* = 57) and pwMS (*n* = 57).

#### Source data

The DICS beamforming analysis with subsequent voxel clustering using the DBSCAN algorithm was conducted prior to connectivity analysis of underlying network architecture. We ran separate DICS beamforming analysis for both conditions (repetition versus switch) for each group (multiple sclerosis versus HC). The ACs derived from the DICS/DBSCAN pipeline were similar within the two groups, but not between groups.

For the HC group, we found three ACs in the theta frequency band located in the left temporo-parietal, right (fronto-)central areas and left occipital regions (Fig. 4A). Regarding pwMS, the AC pattern was different (Fig. 5). First, the number of ACs was lower with two ACs found in the theta frequency band. Second, ACs in pwMS were mainly locally distributed, but less globally. Thereby, one large AC was found encompassing parts of the right temporal, parietal and occipital regions, while the second smaller AC was located on the left hemisphere in the temporo-parietal area. Table 1 displays ACs for both memory-based conditions in HCs and pwMS.

**Figure 4:**
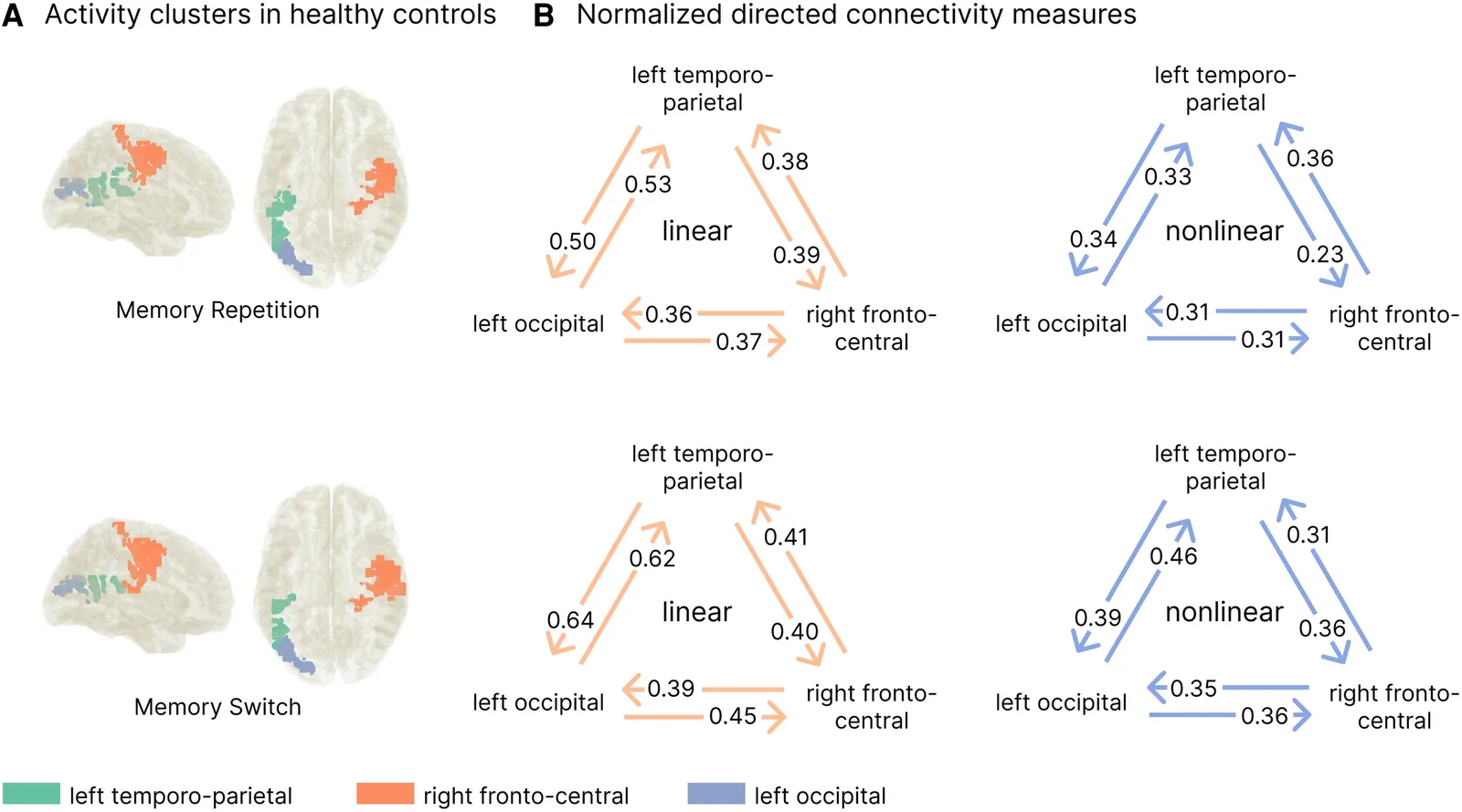
**Effective connectivity network within the theta frequency band in HCs**. The nCREANN algorithm results for the average surrogate-masked normalized connectivity values in the HC group within the theta frequency band are shown. (**A**) The ACs for both memory-based conditions: repetition trials (top) and switch trials (bottom). AC1 (green) represents the left temporo-parietal cluster [BA 21, BA 22/41/42, BA 40, BA 39, BA 43, BA 39/40, BA 1/2/3, BA 13/16], AC2 (orange) corresponds to the right fronto-central cluster [BA 4, BA 1/2/3, BA 9, BA 46, BA 40, BA 43, BA 22/41/42], and AC3 (purple) indicates the left occipital cluster [BA 18, BA 19]. (**B**) Single non-self-connectivities for each condition, with results from LIN = linear (orange) and NONLIN = non-linear (blue) connectivity analyses. All incoming and outgoing connections from and to an AC were not significantly different based on results of paired-sample *t*-tests with *n* = 57 (all *P* > 0.05). AC = activity cluster, BA = Brodmann area.

**Figure 5:**
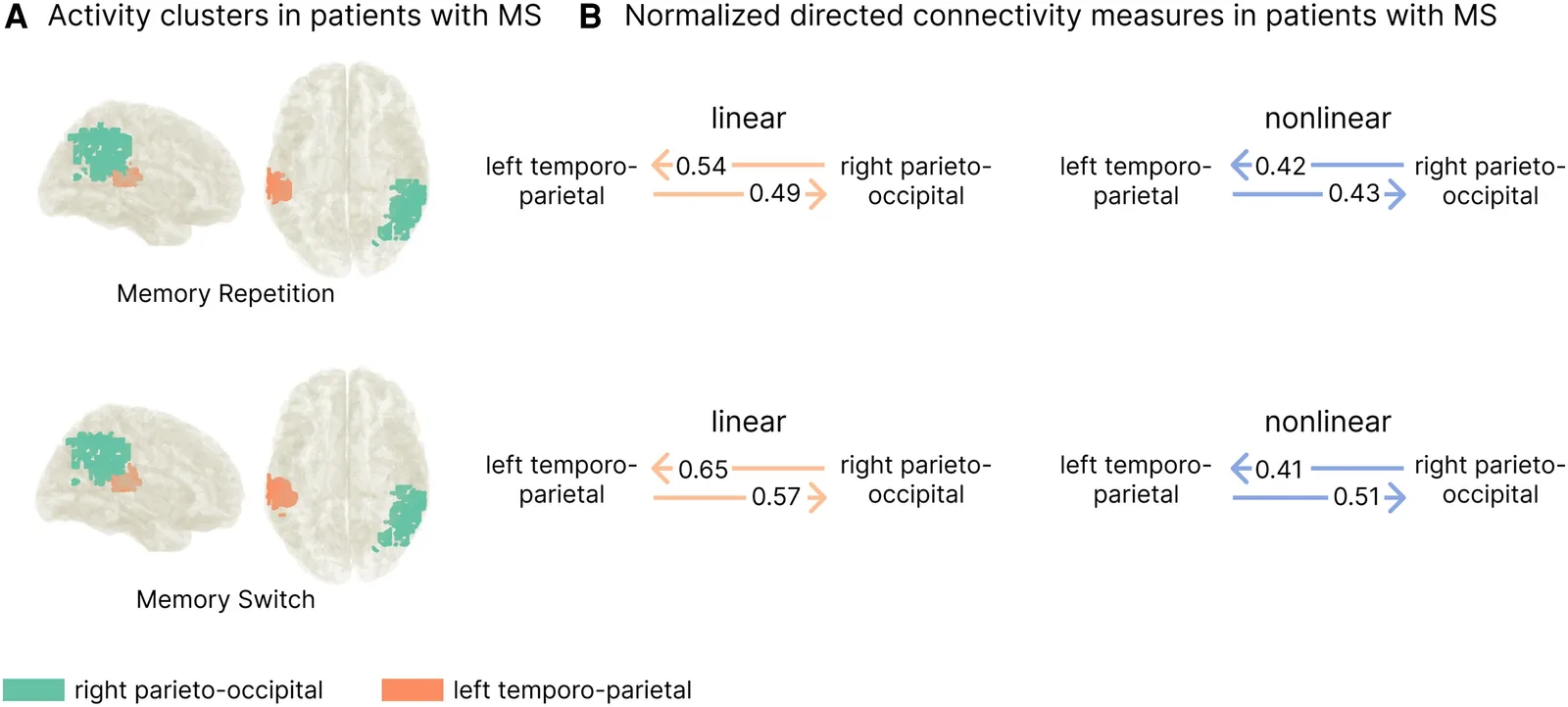
**Effective connectivity network within the theta frequency band in pwMS**. The nCREANN algorithm results for the average surrogate-masked normalized connectivity values in pwMS within the theta frequency band. (**A**) The ACs for both memory-based conditions. AC1 (green) represents the parieto-occipital cluster [BA 39, BA 40, BA 39/40, BA 21, BA 22/41/42, BA 5/7, BA 18, BA 19, BA 1/2/3], while AC2 (orange) corresponds to the left temporo-parietal cluster [BA 22/41/42, BA 40, BA 21, BA 1/2/3, BA 43]. (**B**) Single non-self-connectivities for each condition, with results from LIN = linear (orange) and NONLIN = non-linear (blue) connectivity analyses. All incoming and outgoing connections from and to an AC were not significantly different based on results of paired-sample *t*-tests with *n* = 57 (all *P* > 0.05). AC = activity cluster, BA = Brodmann area.

**Table 1:** ACs in the theta frequency band for both memory-based conditions for HC and multiple sclerosis group

ACs HC group		Brain regions	Brodmann areas
1	Left temporo-parietal cluster	Middle and superior temporal gyrus	BA 21 and BA 22/41/42
		Supramarginal gyrus	BA 40
		Angular gyrus	BA 39
		Rolandic operculum	BA 43
		Inferior parietal gyrus^a^	BA 39/40
		Postcentral gyrus^b^	BA 1/2/3
		Insula	BA 13/16
2	Right fronto-central cluster	Precentral gyrus	BA 4
		Postcentral gyrus	BA 1/2/3
		Middle frontal gyrus	BA 9 and BA 46
		Supramarginal gyrus^c^	BA 40
		Rolandic operculum^b^	BA 43
		Superior temporal gyrus^b^	BA 22/41/42
3	Left occipital cluster	Middle occipital gyrus	BA 18
		Superior occipital gyrus	BA 19

ACs multiple sclerosis group		Brain regions	Brodmann areas
1	Right parieto-occipital cluster	Angular gyrus	BA 39
		Supramarginal gyrus	BA 40
		Inferior parietal gyrus	BA 39/40
		Middle and superior temporal gyrus	BA 21 and BA 22/41/42
		Superior parietal gyrus	BA 5/7
		Middle occipital gyrus	BA 18
		Superior occipital gyrus	BA 19
		Postcentral gyrus	BA 1/2/3
2	Left temporo-parietal cluster	Superior temporal gyrus	BA 22/41/42
		Supramarginal gyrus	BA 40
		Middle temporal gyrus	BA 21
		Postcentral gyrus	BA 1/2/3
		Rolandic operculum^b^	BA 43

^a^Only found in repetition trials.

^b^Only found in switch trials.

^c^Only found in switch trials with more than five voxels.

#### Effective connectivity theta

Figure 4 illustrates the directed connectivity patterns within the theta frequency band for memory-based repetition and switch trials in the HC group. All displayed connections represent significant directed information transfer, as determined through surrogate data testing (see Materials and methods section). In the non-linear network of memory-based repetition trials (Fig. 4B), the descriptively highest connectivity values were found between AC1 (left temporo-parietal cluster) and AC3 (left occipital cluster). Connectivity levels between ACs were relatively balanced in terms of incoming and outgoing connections (i.e. no asymmetry found in directed communication between ACs).

ACs during task switching showed a clearly different pattern for pwMS with only two ACs, namely the right parieto-occipital and the left temporo-parietal cluster (see Section 3.2.2). In Fig. 5, results of the connectivity analysis between the two ACs are shown. Here, the strength of directed communication was similar between both ACs in both conditions and networks (linear versus non-linear).

#### Exploratory correlational analysis

We further took the results of the nCREANN analysis and correlated switch costs in single connectivity measures with behavioural switch costs (e.g. for RTs and hit rates). For pwMS, correlations were also computed with clinical parameters (i.e. disease duration and EDSS scores). To correct for multiple comparisons, a FDR correction was used.

For the HC group, we found only positive correlations in the non-linear theta network between RT switch costs with connectivity switch costs in the directed AC1 → AC2 (*r* = 0.418; *P* = 0.001; *P*_corr_ = 0.009) and AC3 → AC2 (*r* = 0.402; *P* = 0.002; *P*_corr_ = 0.009) connections (see Fig. 6A), indicating higher switch costs with elevated neural connectivity modulations. No correlations were found in the linear theta network, nor with accuracy switch costs overall.

**Figure 6:**
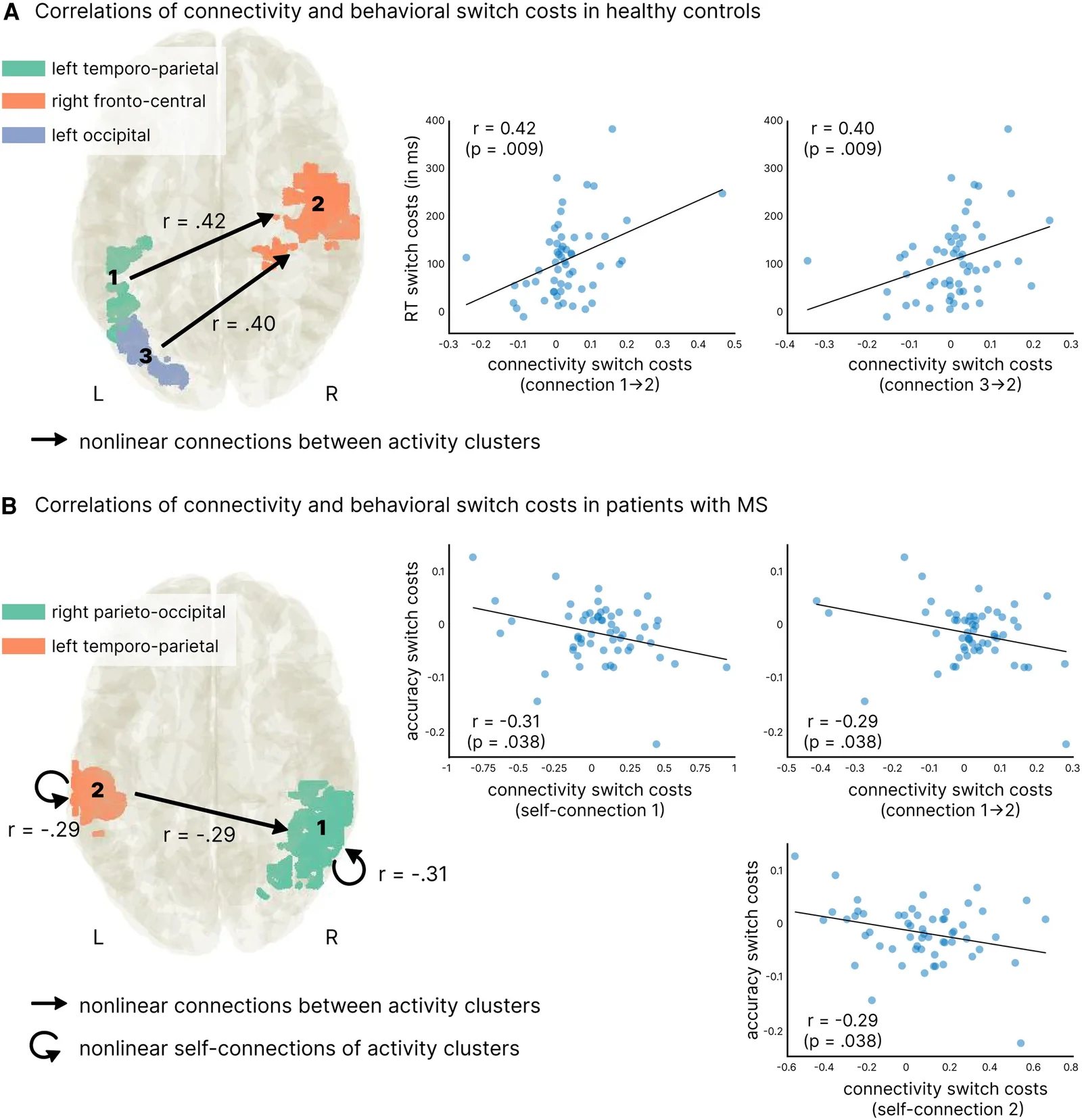
**Correlations between switch costs in neural modulations (directed connectivities derived from nCREANN) and behavioural switch costs (i.e. RT and accuracy switch costs)**. (**A**) Results for the HC group (*N* = 56). On the left side, directed connections between ACs in the non-linear theta network, which correlated with RT switch costs in the HC group, are shown schematically (AC1 = left temporo-parietal cluster, AC2 = right fronto-central cluster, AC3 = left occipital cluster). On the right side, scatter plot results of Pearson’s linear correlation analysis are shown. (**B**) The same pattern for pwMS (*N* = 57) with AC1 = right parieto-occipital cluster and AC2 = left temporo-parietal cluster. Here, also switch costs in self-connectivities of both ACs in the non-linear theta network correlated with accuracy switch costs (indicated by circle-arrow). Each data point represents the relationship between connectivity switch costs (*x*-axis) and behavioural switch costs (*y*-axis) for each participant. *P*-values were FDR corrected. HC group: AC1 → AC2 (*r* = 0.418; *P* = 0.001; *P*_corr_ = 0.009) and AC3 → AC2 (*r* = 0.402; *P* = 0.002; *P*_corr_ = 0.009). pwMS group: AC2 → AC1 (*r* = −0.291; *P* = 0.028; *P*_corr_ = 0.038), self-connection AC1 (*r* = −0.308; *P* = 0.020; *P*_corr_ = 0.038) and self-connection AC2 (*r* = −0.292; *P* = 0.027; *P*_corr_ = 0.038).

Within pwMS, we also found only significant correlations in the non-linear network. Switch costs in hit rates correlated negatively with switch costs in directed connections from AC2 → AC1 (*r* = −0.291; *P* = 0.028; *P*_corr_ = 0.038), and with switch costs in self-connections of AC1 (*r* = −0.308; *P* = 0.020; *P*_corr_ = 0.038) and AC2 (*r* = −0.292; *P* = 0.027; *P*_corr_ = 0.038) (see Fig. 6B). This shows that lower switch costs in hit rates are associated with higher switch costs in neural connectivity. No correlations were found in the linear network, nor with RT switch costs overall. No correlation was found between effective connectivity measures and clinical parameters.

## Discussion

The present study investigated behavioural and neural correlates of adaptive behaviour in pwMS and HCs during a memory-based task-switching paradigm with the focus on network organization in the theta frequency band. Our findings highlight distinct patterns of neural activation and network organization that underlie preserved but less efficient cognitive control processes in pwMS. Behavioural results showed that pwMS were significantly slower compared to HCs in both repetition and switch trials, although accuracy did not differ between groups. These findings suggest that pwMS are able to maintain task performance levels but require more time to execute cognitive operations. The overarching pattern of results with slower RTs and increased error rates in switch trials compared to repetition trials were in line with previous research,^51-53^ also in pwMS.^28^ Interestingly, switch costs (i.e. performance difference between switch and repetition trials), which are considered to mirror task set reconfiguration,^1,56,57^ did not differ significantly between groups for either RTs or accuracy measures, suggesting preserved behavioural task-switching ability (i.e. sufficient task set reconfiguration) in pwMS. This was contrary to findings of Migliore and colleagues,^28^ who found significant differences in switch costs between pwMS and HCs. However, this might be due to methodological differences in the task as the authors used a cue-based task-switching paradigm, while the current study used a memory-based task. Interestingly, even if the groups did not differ in switch costs, EDSS ratings of pwMS were associated with RT switch costs, suggesting that symptom severity, but not disease duration, might be a sensitive measure for capturing adaptive behaviour.

### Altered theta network architecture might reflect compensatory reorganization in people with multiple sclerosis

To investigate underlying network organization during adaptive behaviour, we run a time-frequency analysis with focus on theta band activity, as it is considered to be crucial for the synchronization of brain-wide activity and task set (re)configuration, including stimulus-response binding.^3^ The time-frequency analysis revealed increased theta power in memory-based repetition trials compared to switch trials for both groups. This increase was observed shortly after stimulus onset in central regions and later in central-posterior areas. While this late power increase (i.e. 0.5–1 s after stimulus onset) was left lateralized in HCs, it was rather right lateralized in pwMS. However, no significant between-group differences in theta power were found across conditions, suggesting similar overall theta engagement when considering the power of oscillations. Given that the strength of oscillatory theta responses alone did not explain behavioural performance differences, we further explored underlying theta band generators with a source localization method. In contrast to previous fMRI studies on effective connectivity in neural networks of pwMS,^33,35^ we followed a data-driven approach with no pre-defined seed regions. In contrast to the comparable theta power results, source localization analyses using the DICS beamformer revealed substantial differences in the spatial organization of the theta network during task switching. HCs showed a focused and functionally specialized activation pattern with distinct contributions from the left temporo-parietal, right fronto-central and left occipital regions, while pwMS showed more diffuse and predominantly right-lateralized activity in a broad right parieto-occipital (and a smaller left temporo-parietal cluster). The discrepancy between time-frequency and source activity results can be explained based on the conceptual differences between the two measures. Time-frequency analyses capture oscillatory activity (i.e. power) at the sensor level (i.e. electrodes), while DICS locates the generators of this activity on the source level. The current results show that even if the averaged power at the sensor level, which reflects local oscillatory activity, shows no differences between groups, the underlying network structure can vary significantly (see Figs. 4 and 5). This is also particularly true for measurements of effective connectivity, which are based on source activity results. It should also be noted that increased activation of the source does not necessarily imply increased connectivity, since connectivity measures do not capture local oscillatory activity, but rather interregional synchronization processes independent of local activation. The differences and their possible implications are discussed in the following.

In the HC group, the first AC (i.e. left temporo-parietal cluster) included the temporal gyrus [Brodmann area (BA) 21 and BA 22/41/42], the supramarginal gyrus (BA 40) and the angular gyrus (BA 39), which have been repeatedly associated with stimulus-response mapping,^70-72^ response selection^44,73^ and response-outcome monitoring^74,75^—all processes associated with task set (re)configuration, which are likely mediated by theta band activity.^3,4^ Furthermore, these regions have been involved in memory maintenance^76^ and inhibitory control processes,^77^ which are important for maintaining relevant task set information. Moreover, we found the insular cortex (BA 13/16) and the adjacent Rolandic operculum (BA 43) to be part of this AC, likely further assisting the maintenance and updating of task rules,^78,79^ as well as supporting response inhibition and cognitive conflict resolution.^80-82^ These processes are particularly important during memory-based task switching^83,84^ and are closely associated with theta band activity.^85-87^ Importantly, the insula also helps in estimating cognitive control demands,^88^ a function that corresponds with established views on the role of theta oscillations in cognitive control.^5^ In sum, areas encompassed by the left temporo-parietal cluster (AC1) in the theta network may support task switching by modulating key processes of task set (re)configuration, such as stimulus-response binding and response selection, as well as the maintenance of relevant task sets and rules by adjusting inhibitory resources and cognitive control demands to shield against interferences. The second AC (i.e. right fronto-central cluster) included parts of the ‘cognitive control network’ (Niendam *et al*., 2012) encompassing the right pre- and postcentral gyri (BA 4 and BA 1/2/3) as well as the middle frontal gyrus (BA 9 and BA 46). Traditionally, the pre- and postcentral gyri are mainly associated with motor and sensory functions.^89,90^ However, a recent study by Alahmadi^91^ showed that these regions display functional integrations with several brain networks (such as the dorsal attention or the salience network) and regions associated with cognitive control processes, such as the insula or the supramarginal gyrus, suggesting their importance for tasks that require the integration of sensory and motor information with higher cognitive processes. Additionally, the middle frontal gyrus (as part of the dorsolateral prefrontal cortex) is crucial for working memory processes^92^ and task management by allocating attentional resources^93^ and inhibiting interfering task sets.^17,80,94,95^ Taken together, the second AC comprises regions of the cognitive control network that might aid task switching by integrating sensory and motor information with higher cognitive functions and help focus on relevant task sets by allocating attentional and inhibitory resources. The third AC found in the theta network of HCs was the left occipital cluster, including parts of the middle and superior occipital gyrus (BA 18/19), which are primarily (but not only) involved in visual processing^96^ and may support feature integration of stimulus-driven components during task switching. Additionally, prior studies have linked activation in these regions to attentional load and the allocation of internal attentional resources, reflecting top-down attentional control,^97,98^ and to mechanisms of cognitive interference control.^99^ In sum, the theta network found in the HC group seems to be highly specialized on processes of task set (re)configuration and maintenance and top-down control through attentional and inhibitory processes. Regarding the information flow between ACs, we did not find an asymmetry in connectivity strength, indicating that no AC exerts top-down control over another in the given network. However, connectivity values between the left temporo-parietal (AC1) and the left occipital cluster (AC3) were stronger than connections with the right fronto-central cluster (AC2) in the linear theta network. The strong communication between the left temporo-parietal and occipital cluster without any asymmetry in connectivity strength speaks for a steady exchange of information where attentional resources are constantly required to allow flexible and fast (re)configuration and maintenance of relevant task sets. A slightly weaker connection to areas comprising the motor cortex (i.e. right fronto-central cluster) might reflect a less intense and/or frequent communication regarding the execution of an action compared to a constant communication from the left temporo-parietal and occipital cluster to enable rapid binding and unbinding of task features.

Within pwMS, we also found a left temporo-parietal cluster (AC2); however, this was smaller than in the HC group and consisted of voxels from the temporal gyrus (BA 22/41/42), the supramarginal gyrus (BA 40) and the postcentral gyrus (BA 1/2/3), likely supporting task set (re)configuration and action selection processes^44,70-73^—similar to the HC group. However, a broad posterior right-lateralized parieto-occipital cluster (AC1) encompassing the angular gyrus (BA 39), the temporo-parietal junction [including the supramarginal (BA 40), inferior parietal (BA39/40) and superior temporal gyrus (BA 22/41/42)], the superior parietal gyrus (BA 5/7; also known as precuneus) and the postcentral (BA 1/2/3) and occipital (BA 18/19) regions was evident in the theta network of pwMS during task switching. These regions have been associated with adapted behaviour by facilitating inhibitory (motor) control and interference control, as well as maintenance and updating of task set and response-outcome information for following action plans.^74,75,77,99-103^ Interestingly, no activations was found in the insula or Rolandic operculum (both part of the salience network), nor in frontal areas, which are important sites for attentional resource allocation^93^ and inhibitory control.^17,80-82,94,95^ Overall, the theta network found in pwMS also consisted of regions important for task set (re)configuration, inhibition and memory maintenance. However, important sites for integration processes, cognitive control estimation and attentional allocation, such as the insula or central gyri, were not involved within the theta network. The lack of activation in regions responsible for allocating attentional resources might arise due to several reasons. Results from fMRI-based centrality measures (i.e. total number of connections for each voxel with any other voxel of the brain) reported reduced centrality in the salience and sensorimotor networks on the one hand, but increased centrality in the default mode network in pwMS compared with HCs.^32,104-106^ Further, within the group of pwMS, the same pattern was shown for cognitively impaired compared to cognitively preserved patients.^32^ The lack of activation in the insula might also reflect overall reduced (resting-state) activity in the salience and executive control networks in pwMS.^107,108^ This might also explain why pwMS are slower in the task, since flexible adjustments of cognitive control processes rely heavily on intact functioning of the insula.^12,80,82,88,109^ To date, it is not possible to clearly decide, whether lack of activation reflects reduced activity, altered frequency-specific recruitment or a shift in functional strategy. Nevertheless, the current results suggest that pwMS are less efficient at exchanging information regarding attentional resources and task features, which could lead to longer RTs due to inefficiency. However, intact inhibitory processes related to motor and interference control might explain why pwMS do not also show a loss in accuracy. The engagement of a broad right posterior activation therefore might reflect a compensatory recruitment of alternative brain areas and a functional reorganization that allows pwMS to maintain accuracy (i.e. hit rate similar to HCs) but at higher costs in RTs (i.e. prolonged RTs in pwMS). The right-lateralized parieto-occipital pattern, combined with reduced frontal control network engagement, points to an increased dependence on sensory or bottom-up- rather than top-down-driven strategies for task execution. This shift to a right-lateralized activation could be due to structural damage and/or neural inefficiency in typical task-relevant regions as the left hemisphere seems to be more susceptible to structural damage in pwMS.^110-113^ In the same vein, previous studies reported lateralization effects with regard to cognitive functioning.^113-117^ For example, one study showed correlations between attention and information processing speed with cortical thickness of left temporal gyrus and memory processes,^113^ while another showed that the paced auditory serial addition test (i.e. measure information processing speed, working memory and divided attention) activated preferentially left fronto-parieto-temporal areas.^115^ Moreover, memory disturbances were associated with alterations of the P600 in the left frontal and temporo-parietal areas,^116^ metabolic reductions in left fronto-temporal regions^117^ and the loss of axonal integrity in the left hemisphere.^114^ Preziosa *et al*.^110^ suggest that this asymmetric distribution of damage could be explained by a number of variables, such as the left hemisphere’s increased vulnerability to neuronal and metabolic dysfunction as a result of its ‘overuse’ in right-handers.

However, other explanations are possible, such as maladaptive plasticity, where reorganization of a network is unhelpful or even detrimental or a shift in strategy to fulfil the requirements of a task as pwMS might need to adopt alternative cognitive strategies relying more on right-lateralized processes. Previous research showed that the causes of network reorganization in pwMS are manifold, as connectivity abnormalities can reflect weaker connections, the loss of connections, connection reversal, or the presence of additional (i.e. compensatory) connections.^118^

### Differential brain–behaviour correlations suggest divergent network strategies in people with multiple sclerosis and healthy controls

The previous results focused on functional connectivity (i.e. simultaneous activation) within a network, which has been previously shown to play a critical role in the development of cognitive impairment in pwMS.^32,34^ In the recent years, measures of effective connectivity (i.e. directed communication) also gained more attention but are still scarce. Results from two fMRI studies suggest reduced effective connectivity to be associated with poor cognitive performance.^33,35^ Nevertheless, studies focusing on measures of directed connectivity are rare, especially in the field of neurophysiological research.

In the current study, the nCREANN algorithm was applied to ACs derived from the DICS/DBSCAN pipeline (on time-sensitive EEG data) to investigate directed connectivity within a theta network during task switching. The results showed no asymmetries between directed connectivities of ACs, neither in networks of HCs nor pwMS. In other words, all ACs contributed equally to the organization of the given network, with no AC applying top-down control over another (Figs. 4 and 5). In a further step, we evaluated the relationship between changes in connectivity strength (i.e. connectivity switch costs) and adaptive performance in a correlational analysis. The results revealed differential relationships between neural modulation and behavioural switch costs between groups. Importantly, correlations were only found for connections in the ‘non-linear’ but not linear networks. This suggests a greater sensitivity of non-linear models to biologically plausible mechanisms (e.g. dynamic adjustments, non-additive influences or feedback loops) underlying flexible cognition. Previous research showed that several biological mechanisms (especially brain functioning) are of non-linear nature.^40,119,120^ Prominent examples of this are neural firing patterns (i.e. action potentials) or dopaminergic neurotransmitter modulations. In particular, dopaminergic release influences cognitive functioning in a non-linear inverted U-shaped function, whereby too low and too high dopamine levels result in cognitive dysfunctioning.^121-124^ In cognitive neuroscience, models of decision-making often include thresholds,^125^ which have to be met to initiate an action (e.g. drift-diffusion or attractor landscape models). Thereby, non-linear models offer a possibility to predict these decision thresholds^46^ and are suggested to outperform linear models in the classification of neurological disorders based on functional connectivity metrics.^38,126^

Interestingly, while only directed connectivity modulations in connections from the left to the right hemisphere correlated significantly with behavioural switch costs in both groups, the nature of these associations differed. In the HC group, we found only correlations between behavioural switch costs and modulations in long-range (i.e. global) non-linear connections. More specifically, we found correlations of RT switch costs with modulations in non-linear directional information transfer from the left temporo-parietal (*r* = 0.42) and occipital cluster (*r* = 0.40) to the right fronto-central cluster (Fig. 6). This correlation pattern suggests that especially changes in the transfer rate of information regarding task set properties to executive motor areas are associated with changes in the behavioural outcome. In our study, stronger neural modulations (i.e. greater connectivity switch costs) were associated with greater RT switch costs, suggesting that increased neural engagement between brain regions did not necessarily reflect more efficient processing in HCs. One possible explanation for this pattern might be that enhanced neural modulation could reflect processes triggered by higher work load instead of adaptive resource allocation. Given that a positive value in the neural modulation reflects a higher directed connectivity (or information transfer) in switch than in repetition trials and a stronger top-down control for task set (re)configuration is necessary in switch trials,^1,56^ the positive correlation between neural and behavioural RT switch costs might reflect an additional computational effort to enable increased information flow about properties of a task set towards regions of task execution during task switching, which may cause longer RTs.

In pwMS, however, behavioural switch costs were also linked to changes in local self-connectivity, pointing to a stronger reliance on short-range, intra-regional communication than HCs. In particular, neural modulations in non-linear self-connectivity in the left temporo-parietal AC (*r* = −0.29) and the right parieto-occipital AC (*r* = −0.31), as well as changes in the directed connectivity strength from the left temporo-parietal to the right parieto-occipital AC (*r* = −0.29), correlated with accuracy switch costs, but not RT switch costs. Hereby, it should be noted that a correlation of *r* = 0.3 is considered rather low, so this should be taken into account when interpreting the results, as it could limit their generalizability. Nevertheless, the results provide initial evidence that in pwMS, stronger neural non-linear modulations in/between these ACs are associated with ‘lower’ accuracy switch costs, potentially indicating a more adaptive use of (local) neural resources to support performance or even a compensatory mechanism, which could enhance cognitive flexibility in pwMS. This flexibility may be particularly crucial in the context of task switching, which requires the rapid (re)configuration of task sets (likely supported by the left temporo-parietal AC) and cognitive and motor control processes (supported by the right parieto-occipital AC) as a response to changing goals. Similar to findings from the HC group, enhanced information transfer about current task sets was directed from the left hemisphere via long-range connections to downstream right-lateral regions supporting the maintenance of these task sets and ultimately enable the execution of motor programmes based on the information received.

The presented results support the idea that cognitive performance in pwMS depends more strongly on localized neural dynamics possibly reflecting a reorganization of functional networks due to disease-related damage. This aligns with the concept of a ‘dysconnectivity syndrome’, where disruptions in long-range connections result in a shift towards more local network processing.^24-26^ Importantly, this neural activation pattern suggests that pwMS are not simply showing greater activation due to increased effort, but are instead engaging non-linear regional adjustments that likely reflect a re-allocation of cognitive resources to facilitate goal-directed behaviour.

### Limitations

Due to our focus theta band dynamics during task switching, the presented results cannot be compared one-to-one with other findings of task-switching behaviour, as other frequency bands, such as alpha, beta or gamma, are also important for adaptive behaviour.^3^ Moreover, when looking at previous task-switching literature, the anterior cingulate cortex (ACC) plays a crucial role.^17,20^ Nevertheless, in our study, voxels associated with the ACC were not within the top 3% of all active voxels in a theta band network. Importantly, this does not mean that the ACC is not of importance for task switching. However, given ACCs role in conflict and outcome monitoring^17^ rather than task set configuration, it is possible that an activation might rather occur in other frequency networks, such as alpha, which likely acts as a ‘gate keeper’ for working memory information,^3,127^ or beta, which is considered to support monitoring and maintenance of task sets in the working memory.^3,46,128,129^

One limitation regarding the generalizability of the results to pwMS stems from the fact that current medication (disease-modifying therapy; DMT) was not controlled for. Various DMTs exist for treating multiple sclerosis, targeting different mechanisms of disease progression. Therefore, it is not clear whether patients with different DMTs might show different patterns in network architecture and connectivity.

## Conclusion

The present study offers novel insights into the neural mechanisms of adaptive behaviour in HCs and pwMS, revealing distinct network strategies during task switching. In HCs, enhanced modulations of long-range fronto-parietal connectivities were associated with higher RT switch costs, possibly reflecting increased cognitive demands rather than adaptive processes. In contrast, pwMS relied more strongly on local intra-regional modulations, which is consistent with the idea of a dysconnectivity syndrome in multiple sclerosis. Strikingly, stronger modulations in self-connectivities were associated with better accuracy measures in pwMS, suggesting a compensatory mechanism to support adaptive performance. Notably, only non-linear directed connectivity measures were related to behavioural switch costs, emphasizing the importance of non-linear models for adaptive cognitive processes.

## Supplementary Material

fcag397_Supplementary_Data

## Data Availability

Raw data can be found in the Open Access Repository and Archive from TU Dresden (https://doi.org/10.25532/OPARA-1081). The numerical source data for Fig. 2 can be found in logfiles and tables under the name ‘01_Behavioral data.zip’, and numerical source data for Figs. 3–6 can be found under the name ‘02_Preprocessed EEG data.zip’. Custom code used to process the data can be found in OPARA under the name ‘03_Scripts.zip’.
