# Supplementary material for: Altered network organization with shift to local processing during adaptive behaviour in multiple sclerosis
Source: Brain Commun. 2026 Oct 19;8(5):fcag397. doi: 10.1093/braincomms/fcag397 (PMC13711671; doi:10.1093/braincomms/fcag397)
Supplement: Supplementary file 1 [file fcag397_supplementary_data.pdf]

Supplementary Material

Task and Procedure

Data from six memory-based blocks with a total of 198 trials were used. The target stimuli were digits ranging from 1 to 9 (excluding 5), presented in white font against a black background on a computer screen. The experiment was conducted in a dimly lit room, with participants seated 60 cm away from a 24-inch display screen equipped with an eye-tracking device. To familiarize themselves with the task, participants completed a practice block of 36 trials prior to the main experiment.

The paradigm involved three task rules. The ‘NUM’ rule (short for the German term *numerisch*, meaning “numeric”) required participants to judge whether the digit was smaller or larger than 5. The ‘GER’ rule (*gerade*, meaning “even”) instructed participants to decide whether the digit was even or odd. The ‘SG’ rule (*Schriftgröße*, meaning “font size”) asked participants to determine whether the digit was displayed in a large or small font (font sizes varied between 50 and 80). Each trial began with a (dummy) cue presented 1300 ms before the target digit, which remained visible until the target appeared. Participants should press the left "Ctrl" key if the digit was less than five, had a small font size, or was odd. Conversely, if the digit was greater than five, had a large font size, or was even, participants pressed the right "Ctrl" key. Responses were considered missed if no key was pressed within 2500 ms. After each response, a black screen was displayed for 500 ms, followed by feedback in the form of a “+” (correct) or “–” (incorrect) sign for 500 ms. A jittered feedback-cue interval followed, bringing the total response-cue interval to approximately 2000 ms.

In the memory-based block, participants had to mentally track a fixed sequence of task rules in the following order (NUM, NUM, NUM, GER, GER, GER, SG, SG, SG, NUM, NUM, NUM, …). In the beginning of each trial, a dummy cue (“XXX”) was shown 1300 ms before the target appeared (Figure 1). If participants lost track of the sequence and applied an incorrect rule for three consecutive trials, the regular cues (NUM, GER, or SG) temporarily replaced the dummy cue for the next three trials. Each task rule appeared with equal frequency (i.e., 33.3%). However, due to the fixed order, the switching frequency in the memory-based block was reduced to 33.3%, resulting in 66 switch trials and 132 repetition trials.
